# Detection of Liver Tumour Using Deep Learning Based Segmentation with Coot Extreme Learning Model

**DOI:** 10.3390/biomedicines11030800

**Published:** 2023-03-06

**Authors:** Kalaivani Sridhar, Kavitha C, Wen-Cheng Lai, Balasubramanian Prabhu Kavin

**Affiliations:** 1 Department of Computer Science, Bharathidasan University, Tiruchirappalli 620024, Tamil Nadu, India; 2 Department of Computer Science and Engineering, Sathyabama Institute of Science and Technology, Chennai 600119, Tamil Nadu, India; 3 Bachelor Program in Industrial Projects, National Yunlin University of Science and Technology, Douliu 640301, Taiwan; 4 Department Electronic Engineering, National Yunlin University of Science and Technology, Douliu 640301, Taiwan; 5 Department of Data Science and Business System, SRM Institute of Science and Technology, Kattankulathur Campus, Chengalpattu 603203, Tamil Nadu, India

**Keywords:** tumour prediction, coot optimization algorithm, extreme learning model, deep learning-based interactive segmentation, intensity levels

## Abstract

Systems for medical analytics and decision making that make use of multimodal intelligence are of critical importance in the field of healthcare. Liver cancer is one of the most frequent types of cancer and early identification of it is crucial for effective therapy. Liver tumours share the same brightness and contrast characteristics as their surrounding tissues. Likewise, irregular tumour shapes are a serious concern that varies with cancer stage and tumour kind. There are two main phases of tumour segmentation in the liver: identifying the liver, and then segmenting the tumour itself. Conventional interactive segmentation approaches, however, necessitate a high number of intensity levels, whereas recently projected CNN-based interactive segmentation approaches are constrained by low presentation on liver tumour images. This research provides a unique deep Learning based Segmentation with Coot Extreme Learning Model approach that shows high efficiency in results and also detects tumours from the publicly available data of liver images. Specifically, the study processes the initial segmentation with a small number of additional users clicks to generate an improved segmentation by incorporating inner boundary points through the proposed geodesic distance encoding method. Finally, classification is carried out using an Extreme Learning Model, with the classifier’s parameters having been ideally chosen by means of the Coot Optimization algorithm (COA). On the 3D-IRCADb1 dataset, the research evaluates the segmentation quality metrics DICE and accuracy, finding improvements over approaches in together liver-coloured and tumour separation.

## 1. Introduction

Smart healthcare systems, in particular those that make use of IoMT technology, have benefited from recent expansions in machine learning and computer communication [1,2]. A multimedia-based medical diagnostic system is one of the major demands in the medical healthcare business. Radiologists and doctors can benefit even more from the solutions provided by these intelligently built diagnostic systems [3]. Liver cancer ranks as the sixth most common form of cancer worldwide. The liver is a massive granular organ situated inside in humans. Cirrhosis, acute and chronic liver are on the rise as a direct result of changing lifestyles. As a direct result of these underlying conditions, liver cancer has become the most prevalent form of the disease in many regions [4]. There are more therapy choices available when the cancer is detected early. Liver cancer can only be diagnosed through imaging or radiology testing, as a physical examination is not possible. Imagination tests aid to screen cancer at initial phase and assistance to assess the healthiness of post-treatments [5].

CT scan is an imaging method that is used for both visualizing liver structure and diagnosing/treating liver cancer. Clinicians can use it to assess the liver’s useful volume, tumour size, shape, and location to better tailor a treatment plan. The radiologist needs precise separation of liver tumours to perform these calculations [6]. Usually, the segmentation is performed by evaluating each slice of the CT scan. This process is tedious, laborious and fraught with opportunities for error, which can be categorized into three areas. First and foremost, errors can result from the variation in the appearance, size, and location of tumours from one patient to the next. Second, the line dividing tumour tissue from healthy tissue is not sharply delineated. Third, cancerous tissues can be found in close proximity to other organs. Furthermore, due to the wide range in tumour appearances and densities, liver tumour segmentation is a difficult task [7]. Thus, there is an immediate need for investigate into hepatic tumour segmentation, which improves treatment planning, lessens manual labour, and boosts the success rate of liver cancer procedures by advising oncologists pre- and post-operation.

Over the past few decades, scientists have created numerous interactive and autonomous methods for segmenting liver tumours. The radiologists’ productivity is increased thanks to the development and training of semiautomatic methods made possible by computer-aided designs (CAD). Automatic segmentation of liver tumours is challenging due to several obstacles, such as variations in tumour size, shape, and proximity to adjacent organs [8,9,10]. Incomplete volume effects and increased noise due to CT enhancement are also among the challenges encountered. The automation of liverwort liver tumour division is a challenging study topic with opportunity for development.

In addition, differing encoding procedures have a significant effect on the interactive segmentation performance, making it a practical challenge for CNN-based algorithms to properly encode user interactions. The majority of the literature encodes user relations by translating them, the Gaussian heatmap, or the iso-contours obtained from the user’s clicks [11,12,13]. However, the context of the image is ignored by these encoding techniques. The geodesic distance transform, on the other hand, is sensitive to spatial contrast and spatial smoothness, allowing it to encode user interactions. To handle user-provided interactions, DeepIGeoS employs a transform with a custom-tailored verge. To truncate the resulting geodesic distance map at a suitable threshold value, however, might be a time-consuming process when dealing with objects of varying shapes and sizes [14,15]. In order to enhance the precision and scalability of the segmentation, we hypothesise encoding approach is useful.

We present a new medical picture to address the aforementioned problems, with the ultimate goal of not only achieving high presentation and competence for segmentation of liver tumour. The usage of CNNs is central to our strategy, and the user interface is minimal. For better segmentation from the CNN, we provide a new method based on the Exponential zed Geodesic Distance (EGD) transform for encoding user interactions, which is both context-aware and parameter-free. In addition, we offer an information fusion technique that can fuse new intensity levels with the original segmentation in an effective way. The results demonstrated that our method is significantly better than other interactive segmentation procedures.

The rest of the paper is organised as follows: current methodologies and their details are presented in Section 2, followed by an explanation of the proposed model in Section 3. Section 4 compares the projected model with those currently in use, whereas Section 5 provides a summary and recommendations for future research.

## 2. Related Works

Amin, et al., [16] identified liver disease using optimized generative adversarial network (GAN). The proposed model has three parts including the generation of synthetic images, localization, and segmentation, and the generation of synthetic images is carried out by GAN. The features from input images are extracted using ResNet-50, whereas YOLO-v3 is utilized to localize small liver tumours. Finally, the segmentation task is completed by using a pre-trained model of InceptionResNet-v2. The performance of the proposed method is simulated on the publicly available 3D-IRCADb-01 dataset. The method focused only on segmentation, where the classification of tumours is not performed.

A more effective method of liver and tumour separation from CT images is proposed by Ashreetha, B. [17], who suggests using Gabor Features (GF) in conjunction with three different machine learning algorithms: Random Forest (RF), Support Vector Machine (SVM), and Deep Neural Net (DNN). There should not be any inconsistencies or differences in the GF-generated texture data between different slices of the same organ. In the first, an assortment of Gabor filters is used to extract features at the pixel level. Second, liver segmentation is performed by erasing liver from an abdominal CT image using three distinct classifiers. Finally, the segmented liver picture is subjected to tumour segmentation classifiers. All of the aforementioned classification techniques have been successfully applied to pixel-wise segmentation problems, and the Gabor filter is a good approximation to the human visual system (HVS) of perception.

The primary focus of Ayalew, Y.A., [18] was the application of a deep learning approach to partition liver and tumour from stomach CT scan pictures, with the goal of reducing the time and effort required to diagnose liver cancer. The algorithm’s foundation is the primary UNet design. However, in this research, it reduced the number of filters used in each block and introduced batch normalisation and a dropout layer afterward every block along the shrinking path. The programme achieved a dice score of 0.96 for liver subdivision, 0.74 for liver tumour segmentation, and 0.63 for abdominal CT scan image tumour division. The liver results improved by 0.11 percentage points, whereas the overall liver segmentation results improved by 0.01.

Zheng, R., et al. [19] apply a 4D deep learning model that takes advantage of 3D memory to the issue of lesion segmentation: The projected deep learning approach uses the four-dimensionality of DCE MRI to aid in liver tumour segmentation. A shallow U-net-based 3D CNN module was utilised to extract the DCE phases’ 3D spatial area features, and a 4-layer C-LSTM network module was employed to make use of the phases’ temporal domain information. Networks trained in using multi-phase DCE pictures and multi-contrast images, which capture the dynamic nature of tissue imaging features, are better able to understand the characteristics of HCC. The suggested model outperformed the 3D U-net perfect, the RA-UNet model, and other models used in the ablation research on both internal and external test sets for liver tumour segmentation, with a Dice score of 0.825·0.077. The suggested model performs similarly to the state-of-the-art nnU-Net perfect, which has shown to be superior in a variety of segmentation tests, while also greatly outperforming it in terms of prediction time.

A fully automatic approach to liver segmentation from CT scans is projected by Arajo et al. [20]. The suggested method consists of four primary steps. These processes are procedures. The model used 131 CT LiTS database to test the suggested technique. The average results were 95.45% sensitivity, 99.86% specificity, 95.64% Dice coefficient, 8.28% volumetric overlap error, 0.41% relative volume difference, and 26.60 mm in Hausdorff distance.

For better efficiency in diagnosing liver tumours, Rela, M. [21] adopts a novel model. Histogram equalisation and filtering are used for benchmark and manually gathered datasets. In addition, this research work use adaptive thresholding in conjunction with level set segmentation to carry out liver segmentation. After the liver is segmented, a novel Grey algorithm is applied to segment the tumour using an improved deep learning method called U-Net. On top of that, the GW-CTO algorithm adopts based on a multi-objective function because the length of features increases the difficulty of network training. To further improve upon these carefully chosen features, the GW-CTO algorithm is applied to a Neural Network. With a learning percentage of 85%, the DNN achieved an accuracy of 4.3%, outperforming PSO-HI-DNN by 2.4%, O-SHO-HI-DNN by 5.2%, CTO-HI-DNN by 4.3%, and GWO-HI-DNN by 4.3%.

In [22], Liu et al. offer an AI-based approach for segmenting CT images of liver tumours. K-means clustering (KMC), an AI-based approach, was proposed in this paper and compared to the region growth (RG) technique. Using the Child–Pugh classification system, 120 patients with liver tumours at Research Hospital in Chandigarh, India, were divided into two groups: grade A (58 instances) and grade B (62 cases). The experiments showed that liver tumors had low density on plain CT scans and moderate enhancement during PVP. Liver metastasis was shown to be detectable by CT more often than hepatocellular carcinoma. Results reveal that lipiodol chemotherapy emulsion (LCTE) has a favourable deposition effect in patients with a rich blood type (accounting for 53.14%) and a poor blood type (accounting for 25.73%).

Using CT scan data from the 3D-IRCADb01 dataset, Sabir, M.W. [23] developed a ResU-Net architecture with a very dense number of nodes. ResU-fundamental Net’s feature, the residual block and U-Net architecture, allows for the withdrawal of more information from the input data than a standard U-Net network. Data augmentation, a Hounsfield windowing unit, and histogram equalisation are only some of the picture pre-processing techniques used before used to measure ResU-network Net’s efficiency. The ResU-Net system with residual connections achieved a DSC value of 0.97% for methods, which was much higher than the presentation of state-of-the-art systems for liver tumour documentation.

## 3. A Proposed System

**Figure 1:**
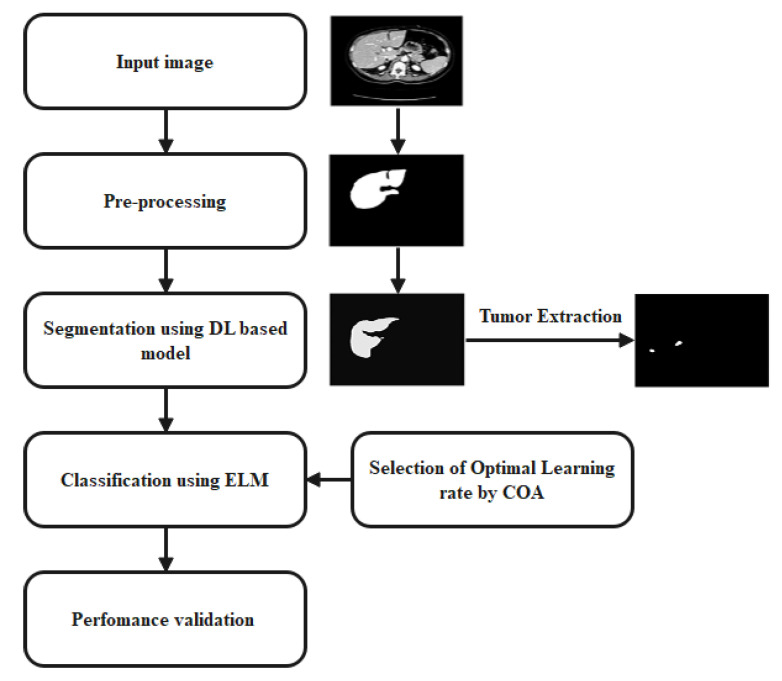
Working flow of the proposed model.

Figure 1 shows the working flow of the proposed model.

### 3.1. Dataset Selection

**Figure 2:**
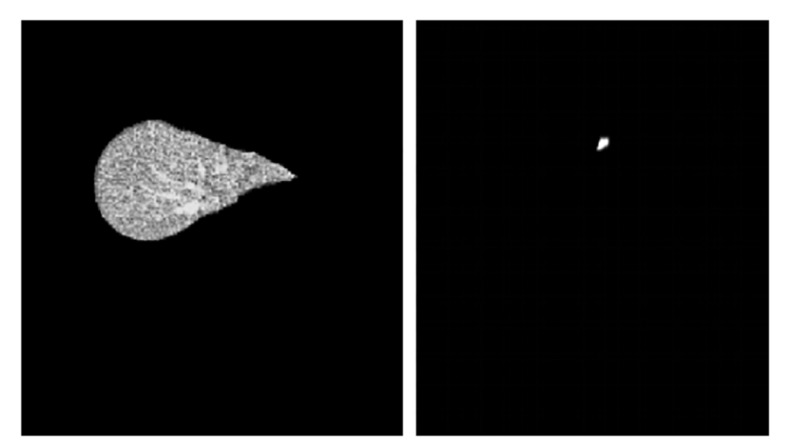
A sample image with a very minute tumour (not visible for the human eye) and its mask.

The proposed approach employs the 3DIRCADb1 dataset [24]. It is a repository of anonymized medical photos and expertly drawn boundaries around organs of interest. The DICOM format is used for all pictures and masks. We used the CT scan pictures from 20 patients found in the 3D-IRCADb1 database, which represents 75% of the tumours in that collection. Each picture is 512 pixels by 512 pixels. The range of slices is anything from 74 to 260, and the slice thickness can be anywhere from 1.25 to 4 mm. Table 1 has all the details. Each scan segment has an appropriate liver mask. Tumour masks, however, are not shared between tissues and can be found in their own dedicated folders. The tumour masks from many scans on different types of tissue were therefore merged into a single folder for computational convenience. An initial sample of 17 patients is used as a training set for the proposed approach, whereas the remaining sample of three patients serves as a test set. Due of the complexity of the liver and its malignancies, this dataset is being investigated for training and evaluation. It has many tumours that cannot seen by the naked eye, and the tumours have a similar contrast to the liver; more specifically, their HU values are practically identical (Figure 2).

Because of these challenges, we decided to investigate this dataset using deep learning techniques.

### 3.2. Data Augmentation

**Figure 3:**
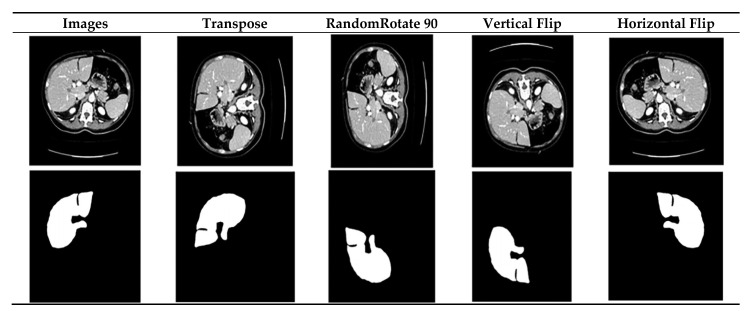
Effect of augmentation procedures on the unique copy.

Data inadequacy is a major obstacle for deep learning representations used to medical data. Not enough data points are available in the datasets to satisfy the needs of the data-hungry deep learning algorithms. Therefore, data augmentation is a popular and viable option for expanding the sample size. When augmentation is required, the Augmentation API37 is used. This package, written in Python, makes it easy to add enhancements to data. To find the most versatile additions, we analysed all of them and settled on four: 90° rotation, transposition, horizontal flip, and vertical flip. Due to the limited size of the dataset, there is a possibility of the network being overfit. Select online augmentation strategies are utilised to prevent this situation from occurring. As a first step, the training data is partitioned into exercise and validation sets. Next, augmentation methods are functional to each slice, and lengthways with their corresponding ground truth (Figure 3).

### 3.3. Data Normalization

The primary goal of data normalisation is to convert a dataset’s numeric columns to a more human-readable scale while maintaining the original data’s range variability. Data normalization is not mandatory in ML, but becomes important when the data features a wide range of values. Through the use of data normalisation, we can ensure that each row only stores the data it needs to and eliminate any potential for data duplication. The normalised data takes up far less room than the alternative. The key worry for data collection and storage is, of course, a lot of memory. Data normalisation is employed to lessen storage needs. There is a flexible database architecture in place. If the same piece of information is stored more than once, data redundancy is committed. Regulating values slow on multiple scales to a theoretically shared scale, frequently before averaging, is what is meant by the term “data normalisation.” First, we assume that $EF_{st}^{\;}$ is a data attribute, and then we define ef1 and ef2 as the minimum and maximum normalised values, respectively. Standardization Equation (1) displays the mathematical form of data normalisation.

(1) $$EF_{st}^{normalization}=\left(ef1-ef2\right)*\frac{\left(EF_{st}-EF^{min}\right)}{\left(EF^{max}-EF^{min}\right)}+ef1$$

In Equation (1), the attribute of the data to be standardised is marked by $EF_{st}$, the normalised data is denoted by $EF_{st}^{normalization}$, the supreme attribute value connected to each record is signified by $EF^{max}$, and $EF^{min}$ mentions to the least quality value connected to apiece record.

### 3.4. Segmentation

The segmentation process has two distinct phases. To begin, the user clicks a few times in the vicinity of the target object’s (input image) border (i.e., its interior margin point). With these points, a loose bounding box can be inferred for use in cropping the input image. Our suggested EGD transform takes the cropped image as input and uses the user-supplied inner margin points to generate a cue map. The cropped input image and the cue map are fed into a CNN to produce a rough segmentation. In the additional phase, the user clicks to highlight mis-segmented areas, and we apply Information Fusion and Graph Cuts to refine the result (IF-GC). The refinement step can be repeated as many times as necessary throughout testing to obtain a satisfactory end product. Our approach requires only a limited sum of training data and can be used immediately to segment new objects without the requirement for additional annotations or fine-tuning.

#### 3.4.1. Inner Margin Points

Interactive cues such as scribbles, bounding boxes, and their combinations are commonly used, but due to variations in individuals and imaging protocols, it can be challenging and time-consuming to identify the precise extreme points in medical images, increasing the workload on the user. We propose using inner margin points as user interactions to work around these restrictions, where the user needs only a few clicks to completely enclose the target area. For organs with complex and irregular shapes, points can provide more shape information, which only uses an additional point. In addition, it can be challenging for users to place clicks accurately on the object boundary, even during testing. Therefore, it is easier to adjust inaccurate clicks by moving them closer to the object boundary. A transform of the exponentialized geodesic distances between these locations along the interior border can provide a good approximation of the saliency map of the target object. As a result, interior margin points may be useful for directing CNNs as they deal with a wider variety of unseen objects.

Every object’s internal boundary points were simulated automatically during training using the ground truth mask and edge detector. The inner margins are calculated using two criteria: In the first place, these points need to be positioned within the object and close to its edge. Second, the entire object space should fit within a tranquil bounding box computed from these locations. As a result, we use a two-stage simulation to mimic user interactions with a training image. (1) A small number of points are picked near the extremes of the target object on the ground truth boundary to make sure the relaxed bounding box encompasses the entire region of interest. Then, to provide further insight into the target’s overall form, we pick n random points from the remaining border points. (2) In step 1, we move all these points towards the inner side of the border to simulate genuine user clicks which may not be entirely accurate. Because users are expected to position the inside margin points inside the border, we rotated the simulated points inwards to reflect this need. Then, the determined bounding box is expanded outward by a few pixels/voxels to incorporate the surrounding area. In the testing phase, the user must supply the inner margin points in a way that complies with the aforementioned conditions. Users’ interactions determine a loose boundary box, which is then enlarged somewhat to incorporate further context.

#### 3.4.2. Exponentialized Geodesic Distance Transform

Coding user interactions efficiently is essential for CNN-based interactive approaches. An ideal encoding technique would incorporate image context and work seamlessly with convolutional neural networks (CNNs) without needing any custom-designed parameters. Although these benefits are desirable, they are not shared by other existing interaction encoding approaches such as the Euclidean distance transform, the Gaussian heatmap, the iso-contours, or the geodesic distance transform. We suggest a context-aware approach to this issue: a hybrid of the geodesic distance and exponential transforms.

Imagine $S_{s}$ is the collection of training-stage simulated interior margin points or user-provided test-stage inner margin points. Assuming that I is a pixel or voxel in the contribution picture I, we can write the nameless I to $S_{s}$ as

(2) EGDi,Ss,I=ej∈Ssmin−Dgeoi,j,I

(3) Dgeoi,j,I= p∈pi,jmin ∫01‖∇Ipn.vn‖ dn

p is a feasible path with parameters n [0; 1], and $P_{\left(i,j\right)}$ is the usual of all pathways between pixels/voxels I and j. The tangent unit vector is defined as $n\in \;\left[0;\;1\right].\;v\left(n\right)=p\prime \left(n\right)/\left|\left|p^{\prime }\left(n\right)\right|\right|$. In this paper, the EGD is described for scalar images, but it may be easily generalised to vector-valued images. Specific instances of cue maps created using various encoding techniques applied to some interior margin points are displayed. It is clear that maps are between the foreground and contextual. This means it could provide the CNN a better initial segmentation result by providing additional shape, location, and context information.

#### 3.4.3. Early Segmentation Constructed on Cue Map and CNN

In this research, we tackle the problem of dealing with both visible and hidden objects in a wide variety of image formats by developing a universal and efficient framework. For this reason, our system is not restricted to any one particular CNN architecture. We employ 2D-UNet and 3D-UNet with certain modifications to show off its potential. To strike a better balance between performance, memory cost, and time consumption, we halve the number of feature channels and swap out the batch normalising layers for instance normalisation layers, which can better adapt to various image types. As mentioned in Section 3.4.1, all inside margin points and relaxed leaping boxes during training. To feed the image into the CNN, all the points inside the margin are turned into a cue map. The user will be requested to input inside margin points for a target in the testing phase. A preliminary segmentation result can then be provided by the CNN. We use a refinement stage that fuses data from the first segmentation with data from further user clicks to fix the mis-segmentation, as will be seen in Section 3.4.4.

#### 3.4.4. Refinement Constructed on Data Fusion among Preliminary Segmentation and Extra User Clicks

Refining a preliminary segmentation is crucial for interactive segmentation based on deep learning. Traditional approaches either need to fine-tune the perfect for a particular image or require an extra model for refinement. These methods of refinement need a lot of resources, especially time and memory, and they are not yet ready for usage with unseen objects. CNN’s prediction was additionally refined automatically by CRF and these CRF-based refining approaches were not intended for interactive segmentation. In contrast to existing approaches, we present a unique strategy for information fusion among original segmentation and further user connections that yields improved generalisation to unknown items without the need for extensive re-training.

During the refinement phase, the user is prompted to click on areas of the image that were incorrectly segmented as foreground or background. We re-apply the projected EGD transformation to generate two more interaction-derived cue maps, which we then use to efficiently encode the newly discovered interactions: $E^{f}$ and $E^{b}$ are EGD-derived cue maps that influence the user’s foreground and background clicks to hone down on certain features. Please take note that in the refinement stage, we do not simply recycle the original EGD map we received in the first phase, but instead use a combination and refinement clicks to determine the new EGD maps. Similarity clicks are represented by the values of $E^{f}$ and $E^{b}$, which fall in the range [0; 1]. The initial foreground and background probability map that is obtained by CNN are denoted as $P^{f}$ and $P^{b}$ and a pixel/voxel is defined as i in the input image I, respectively. It is suggested that $P^{f}$ and $P^{b}$ be fine-tuned in accordance with $E^{f}$ and $E^{b}$ using the information fusion technique. When pixel I is near the refinement clicks, we want to automatically emphasise $E^{f}$ and $E^{b}$; otherwise, $P^{f}$ and $P^{b}$ will be left unaltered. Probabilities of being in the forefront ($R_{i}^{f}$) or background $R_{i}^{b}$) at pixel I as defined by the user, are defined here as

(4) $$E_{i}^{f}=\frac{e^{-D_{i}^{f}}}{e^{-D_{i}^{f}}+e^{-D_{i}^{b}}}$$

(5) $$E_{i}^{b}=\frac{e^{-D_{i}^{b}}}{e^{-D_{i}^{f}}+e^{-D_{i}^{b}}}$$

(6) $$R_{i}^{f}=\left(1-a_{i}\right)*P_{i}^{f}+a_{i}*E_{i}^{f}$$

(7) $$R_{i}^{b}=\left(1-a_{i}\right)*P_{i}^{f}+a_{i}*E_{i}^{b}$$

(8) $$a_{i}=e^{-\min\left(D_{i}^{f},D_{i}^{b}\right)}$$

for some weighting factor $\alpha_{i}\;\in \left[0;\;1\right]$ that changes on its own and dynamically. When I is near the clicks, $R_{i}^{f}$ ($R_{i}^{b}$ is more affected by $E_{i}^{f}$ ($E_{i}^{b}$), and I is nearer to 1.0. If we receive no input for the foreground (background), we will use a fixed value for $D_{i}^{f},D_{i}^{b}$. So, if we call the foreground clicks “$C^{f}$” and the background clicks “$C^{b}$,” then the total number of clicks is $C=C^{f}\cup C^{b}$. Let us call this pixel’s user-supplied label $c_{i}$; if it’s in the clicks, then $c_{i}=1$ if $i\in C^{f}$ and $c_{i}$ = 0 if i $\in C^{b}$. For the final, refined segmentation, we use a (CRF) that incorporates both $R^{f}$ and $R^{b}$:

(9) $$E=\sum_{i}\phi \left(y_{i}|I\right)+\lambda .\sum_{i,j}\psi \left(y_{i},y_{j}|I\right)$$

(10) $$\mathrm{Subject}\;\mathrm{to}:\;y_{i}=c_{i}\;\mathrm{if}\;i\in C$$

where ϕ then ψ are the energy terms, correspondingly. λ postulates a comparative weight among ϕ and ψ. In paper:

(11) $$\phi \left(y_{i}|I\right)=-\left(y_{i}\log\left(r_{i}\right)+\left(1-y_{i}\right)\log\left(1-r_{i}\right)\right)$$

(12) $$\psi \left(y_{i},y_{j}|I\right)\propto exp\left(-\frac{{\left(I_{i}-I_{j}\right)}^{2}}{2\sigma^{2}}\right).\frac{1}{dist_{ij}}$$

$r_{i}$ signifies the value of pixel i in $R^{f}$, and $y_{i}=1$ if I belong to the forefront and 0 then. $I_{i}$ and $I_{j}$ mean pixel i and j in image I, respectively. $dist_{ij}$ is the Euclidean distance among pixel/voxel i and j. σ is a limitation to controller. The CRF problem expressed in Equation (8) is shown to be submodular and solvable by Graph Cut using the max-flow/min-cut algorithm.

#### 3.4.5. Gradient Computation

The following equations are then used to determine the gradients at each pixel in the image:

(13) $$dx=I\left(x+1,y\right)-I\left(x,y\right)$$

(14) $$dy=I\left(x,y+1\right)-I\left(x,y\right)$$

(15) $$\theta \left(x,y\right)=tan^{-1}\left(\frac{dy}{dx}\right)$$

#### 3.4.6. Dividing the Input Image into Cells and Blocks

**Figure 4:**
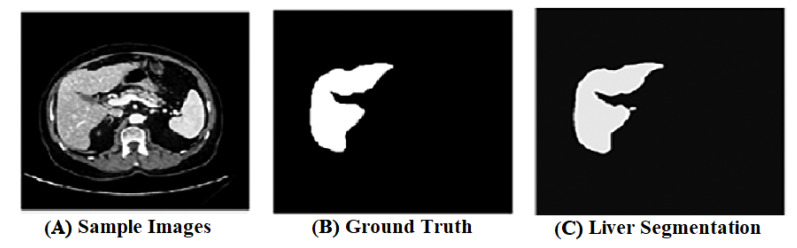
Sample Output for liver segmentation. (**A**) Sample image; (**B**) Ground Truth; (**C**) Liver Segmentation.

**Figure 5:**
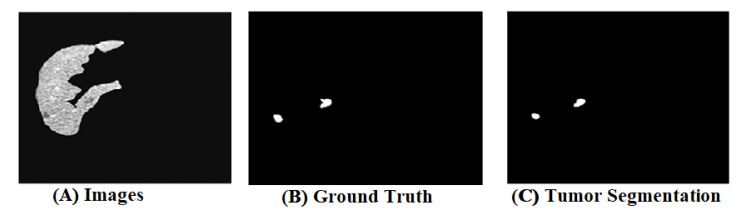
Tumour Segmentation. (**A**) Image; (**B**) Ground Truth; (**C**) Tumor Segmentation.

The gradient image generated at the end is divided into 105 squares by tiling it into 8 × 8-pixel cells and pushing a 16 × 16-pixel window across the cells. This window covers four adjacent cells at a time, and the cells in each group of four are combined to form a block. This block sections are created using this HOG-style image partitioning technique can be observed in the resulting image of size 64 × 128 pixels. It is essential for further procedures including feature extraction. Figure 4 presents the sample images for ground truth and liver segmentation. Figure 5 presents the tumour segmentation.

#### 3.4.7. Construction of the Histogram of Concerned with Gradient Using Selective Sum of Histogram Bins

A histogram of the gradient’s direction is built for each block. To achieve this, the orientation angles of each pixel are used to cast a vote for placement in one of a set number of histogram bins. More bins will be used to extract more specific orientation information from the image, but more features will be generated as a result.

Varying sections of the image use a different sum of histogram bins to minimise the feature size while still preserving the critical information in the feature. For regions that could be part of a human figure, a higher sum of histogram bins is used to extract characteristics, whereas a lesser number of bins is employed for the remaining regions.

An average image is built using 739 positive training samples to locate possible human body parts. The shaded blocks’ features are extracted using a larger sum of histogram bins, whereas the remaining blocks’ characteristics are extracted using a smaller sum of histogram bins. In practise, the optimal values for the high and low number of bins to employ are found by trial and error.

### 3.5. Classification Using Extreme Learning Machine

A neural network enhanced by the gradient method, an ELM. Whereas other gradient learning methods require frequent iterations to maintain optimal network parameters, ELM merely requires a random initialization of the connection weights among the input and output layers and the bias parameters in the hidden layer prior to data training. Once the hidden layer’s neuron count is determined, a single optimal key can be obtained. This type of learning algorithm can speed up the learning process and decrease the amount of time spent analysing data.

In the construction of the ELM, there are N random examples $\left(X_{i},t_{i}\right)$, where $X_{i}\;=\;[x_{1i},x_{2i},\ldots ,x_{ni}{]}^{T}\in \;R^{n},\;t_{i}=[t_{1i},t_{2i},\ldots ,t_{qi},\ldots ,t_{mi}{]}^{T}\in \;R^{m}$. A single-hidden-layer neural uttered as

(16) $$\sum_{i=1}^{L}\beta_{i}g\left(W_{i}.X_{j}+b_{i}\right)=o_{j},j=1,\ldots ,N$$

where, $g\left(x\right)$ is the start of the hidden neuron, $W_{i}\;=\;{\left[w_{i,1},w_{i,2},\ldots ,w_{i,n}\right]}^{T}$ is the input weight, $\beta_{i}$ is the output weight layer expressed as

(17) $$\sum_{j=1}^{N}\|o_{j}-t_{j}\|=0$$

Specifically, there are proper $\beta_{i}$, $W_{i}$, $b_{i}$ which can content the Equation (4). This formula can be signified as

(18) Hβ=T

where H is the hidden layer’s output matrix, is the hidden layer’s output weight, and T is the predictable output. Finding the optimal values for ${\hat{w}}_{i},\;{\hat{b}}_{i},\;{\hat{\beta }}_{i}$ is equivalent to solving the minimal cost function, which is what is required to network.

(19) $$E=\sum_{j=1}^{N}{\left[\sum_{i=1}^{L}\beta_{i}G\left(w_{i}.X_{j}+b_{i}\right)-T_{j}\right]}^{2}$$

A universal classifier that can accomplish, the aforementioned ELM is exactly what is needed. Binary and multiclass classification are both possible with ELM in the field of classification. The learning pace can be drastically increased without the necessity for iterative learning. Although ELM has universal approximation, it requires considerable hidden nodes to ensure a good generalization performance, which is prone to overfitting. This makes the prevention of overfitting an urgent problem of ELM. As a regularization method for fully connected networks, Dropout [25] and DropConnect [26] can effectively prevent overfitting [27]. The steps of the ELM algorithm are as follows [28].

**Step 1:** Give training set $\psi =\left(x_{i},t_{i}\right)|x_{i}\in R^{n},t_{i}\in R^{m},i=1,\ldots ,N$, activation function $G\left(x\right)$, and the sum of hidden neuron L.

**Step 2:** Arbitrarily assign the value of the input weight $w_{i}$ and the bias $b_{i}$. The optimal solution for weight is identified by COA, which is described in Section 3.5.1.

**Step 3:** Compute the hidden layer production matrix H.

**Figure 6:**
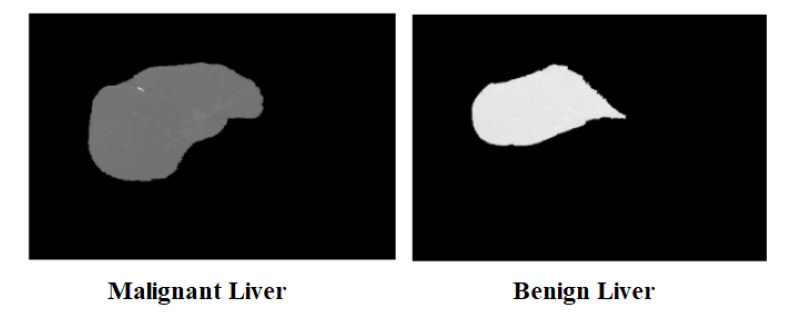
Sample label output of ELM classifier for input image.

**Step 4:** Compute the output weight β: β=H†T, where H† is the Moore–Penrose widespread inverse matrices H. Figure 6 presents the sample label output of input image, which is obtained from the classifier ELM. The binary output “0” and “1” is obtained by ELM, which is labelled by the input image and shown in Figure 6.

#### 3.5.1. Coot Optimization Algorithm

In [29], a new optimization algorithm called the COA is introduced, and its design is based on the behaviour of coot birds. COA makes an effort to imitate the flock’s behaviour as a whole. A few coots floating on the water’s surface are in charge of guiding the flock. From what we can tell, they exhibit four distinct behaviours: random wandering, moving in chains, shifting their positions relative to the group leaders, and guiding the pack to the best possible location. These behaviours cannot be implemented without a mathematical model.

Initial conditions include the generation of a random population of coots. Let us pretend we have a D-dimensional problem that needs solving. Using Equation (20), we can construct a population of *N* coots.

(20) $$PosCoot\left(i\right)=random\left(1,D\right)\times \left(UB-LB\right)+LB,\;i=1,2,.,N$$

Based on the upper boundaries *UB* and lower bounds *LB* calculated for each dimension, Equation (20) generates a uniform distribution of coot positions in a higher-dimensional space. The coots cannot go beyond or below these levels of protection. A specified fitness function, as shown in Equation (21), is applied to this initial random population as well.

(21) $$F\left(i\right)=Fitness\left(PosCoot\left(i\right)\right),\;i=1,2,\ldots ,N$$

First, a random location is generated using Equation (22) to represent the unpredictable behaviour of coots. Second, we calculate the coot’s new location using Equation (23).

(22) $$R=random\left(1,D\right)\times \left(UB-LB\right)+LB$$

(23) $$PosCoot\left(i\right)=PosCoot\left(i\right)+A\times RN2\times \left(R-PosCoot\left(i\right)\right)$$

In Equation (23), *RN*2 is an arbitrary number between zero and one. Both *A* and *B* are calculated using Equation (24):

(24) $$A=1-\left(T\left(i\right)\times \frac{1}{IterMax}\right),B=2-\left(T\left(i\right)\times \frac{1}{IterMax}\right)i=1,2,\ldots ,IterMax\;$$

$T\left(i\right)$ is the current repetition in Equation (24), and *IterMax* is the maximum sum of iterations allowed. The average location of two coots is used to bring one closer to another in order to accomplish chain movement, as shown in Equation (25).

(25) $$PosCoot\left(i\right)=0.5\times \left(PosCoot\left(i-1\right)+PosCoot\left(i\right)\right)$$

Coots also choice a leader coot and shadow them using Equation (26):

(26) $$L_{ind}=1+\left(iMOD\;N_{L}\right)$$

$N_{L}$ is the parameterized number of leaders, and Lind is the leader index, in Equation (19). To go along with this, we also have the concept of a probability, denoted by *p*. When all else fails, we turn to Equation (27)’s rules for assigning leadership roles.

(27) $$LeaderPos\left(i\right)=\left\{\begin{array}{c} B\times R3\times \cos\left(2R\pi \right)\times (gBest-LeaderPos\left(i\right)+gBest\;\;R4<P)\\ B\times R3\times \cos\left(2R\pi \right)\times (gBest-LeaderPos\left(i\right)+gBest\;\;R4\geq P \end{array}\right\}$$

Because *gBest* is the current worldwide best and is 3.14, *R*3 and *R*4 are arbitrary statistics in the interval [0, 1]. The COA’s pseudocode is provided in Algorithm 1 [30].

**Algorithm 1:** Pseudocode of the COA.


$Initialize\;the\;first\;population\;of\;coots\;randomly\;or\;weights\;parameters\;of\;ELM\;by\;Equation\;\left(20\right)$

Initialize the termination or stopping condition for optimal solution, probability p, number of leaders and number of coots 
Ncoot=Number of coots−Number of leaders 
Random selection of leaders from the coots 
Calculate the fitness of coots and leaders 
Find the best coot or leader a the global optimum while the end criterion is not satisfied 
$Calculate\;A,B\;parameters\;by\;Equation\;\left(24\right)$ 
If rand<P 
R, R1, and R3 are random vectors along the dimensions of the problem 
Else 
R,R1, and R3 are random number 
End 
For i=1 to the number of the coot 
$Calculate\;the\;parameter\;of\;K\;by\;Equation\;\left(26\right)$ 
If rand>0.5 
$Update\;the\;position\;of\;the\;coot\;by\;Equation\;\left(27\right)$ 
Else 
If rand<0.5 i~=1 
$Update\;the\;position\;of\;the\;coot\;by\;Equation\;\left(27\right)$ 
Else 
$Update\;the\;position\;of\;the\;coot\;by\;Equation\;\left(25\right)$ 
End 
End 
Calculate the fitness of coot 
If the fitness of coot 
$If\;the\;fitness\;of\;coot<the\;fitness\;of\;leader\;\left(k\right)$ 
$Temp=Leader\left(k\right);leader\left(k\right)=coot;\;coot=Temp;$ 
End 
For number of Leaders 
$Update\;the\;position\;of\;the\;leader\;using\;the\;rules\;given\;in\;Equation\;\left(27\right)$ 
If the fitness of leader<gBest 
$Temp=gBest;\;gBest=leader;\;leader=Temp;\left(update\;global\;optimum\right)$ 
End 
End 
Iter=iter+1; 
End 


Postprocessor results




## 4. Results and Discussion

### 4.1. Segmentation Analysis

**Dice Similarity Coefficient**

DSC, or Dice resemblance coefficient, is commonly used to compute the similarity between two samples. In this research work, this performance measure determined the overlap among two binary masks. It can be mathematically defined as the size of the overlay between two segmentations alienated by objects. The provided range of DSC is usually from 0 (no overlap) to 1. *DSC* is calculated using the following equation:

(28) $$DSC=\left(\frac{2TP}{2TP+FP+FN}\right)$$

**Jaccard Similarity Coefficient**

JSC gives binary mask values precisely. It is also defined as the ratio of similarity and diversity of samples used in experimentation. In mathematical terms, it is the relation of the connexion between two binary covers with their union. *JSC* is calculated according to the equation given below:

(29) $$JSC=\frac{TP}{TP+FP+FN}$$

**Accuracy**

Accuracy is one of the most significant presentation measures that determine the efficiency and effectiveness of any model. Accuracy represents total number of samples [31].

**Symmetric Volume Difference**

SVD provides the alteration of the segmented images from the ground truth. If the value of SVD is zero, it represents a promising resultant segmentation value. The equation determines how to calculate *SVD*, where *DSC* is the Dice similarity coefficient:

(30) $$SVD=\left(1-DSC\right)$$

**Sensitivity**

The properly identified proportion of true positives is measured using sensitivity [32].

**Specificity**

The correctly identified proportion of true negatives is measured using specificity [32].

The existing technique GW-CTO [21] is implemented in our system and results are averaged in Table 2.

Based on the accuracy analysis, the proposed model achieved 93%, whereas the existing GW-CTO method achieved 92%. Additionally, the proposed model obtained a Dice score of 77.11%, which is higher than the existing technique that had a Dice score of 67%. In addition, GW-CTO achieved 56% of Jaccard, 70% of specificity and 64.8% of sensitivity, where the proposed model achieved 67.8% of Jaccard, 79.16% of specificity, and 76.03% of sensitivity. In order to test the generability of the proposed model, its effectiveness is tested with other dataset called Silver07 and it has a lateral resolution of [0.56, 0.8] mm and a z-axis resolution of [1, 3] mm. All sizes of tumours, metastases, and cysts are represented. The central venous phase of all datasets included contrast-enhanced drugs. There are a total of 30 sets in the dataset; 20 for training and 10 for testing. Table 3 presents the comparative analysis of the proposed model with existing technique on second dataset.

In the analysis of accuracy, the proposed model achieved 92% and existing model achieved 91%, where the reason for better performance is the proposed model focused on liver and tumour segmentation using various models. When these models are tested with the Dice score, the existing technique has 70.7% and the proposed model has 77.54%. The sensitivity and specificity of the proposed model is 77.51% and 80.36%, where the existing techniques achieved only 66.6% and 73.5%.

### 4.2. Classification Analysis

**Evaluation metrics**

**Accuracy:** “ratio of the observation of exactly predicted to the whole observations”. This is exposed in Equation (31).

(31) $$T^{accuracy}=\frac{\left(Tr^{p}+Tr^{n}\right)}{Tr^{p}+Tr^{n}+Fa^{p}+Fa^{n}}$$

Sensitivity: “the number of true positives, which are recognized exactly”.

(32) $$Se=\frac{Tr^{p}}{Tr^{p}+Fa^{n}}$$

Specificity: “the number of true negatives, which are determined precisely”.

(33) $$Sp=\frac{Tr^{n}}{Fa^{n}}$$

Precision: “the ratio of positive observations that are predicted exactly to the total number of observations that are positively predicted”.

(34) $$Pr=\frac{Tr^{p}}{Tr^{p}+Tr^{p}}$$

*FPR*: “the ratio of count of false positive predictions to the entire count of negative predictions”.

(35) $$FPR=\frac{Fa^{p}}{Fa^{p}+Tr^{n}}$$

*FNR*: “the proportion of positives which yield negative test outcomes with the test”.

(36) $$FNR=\frac{Fa^{n}}{Tr^{n}+Tr^{p}}$$

*NPV*: “probability that subjects with a negative screening test truly don’t have the disease”.

(37) $$NPV=\frac{Fa^{n}}{Fa^{n}+Tr^{p}}$$

*FDR*: “the number of false positives in all of the rejected hypotheses”.

(38) $$FDR=\frac{Fa^{p}}{Fa^{p}+Tr^{p}}$$

*F*1 *score*: It is distinct as the “harmonic mean between precision and recall. It is used as a statistical measure to rate performance”.

(39) $$F1score=\frac{Se.Pr}{Pr+Se}$$

*MCC*: It is a “correlation coefficient computed by four values”.

(40) $$MCC=\frac{Tr^{p}\times Tr^{n}-Fa^{p}\times Fa^{n}}{\sqrt{\left(Tr^{p}+Fa^{p}\right)\left(Tr^{p}+Fa^{n}\right)\left(Tr^{n}+Fa^{p}\right)\left(Tr^{p}+Fa^{n}\right)}}$$

**Figure 7:**
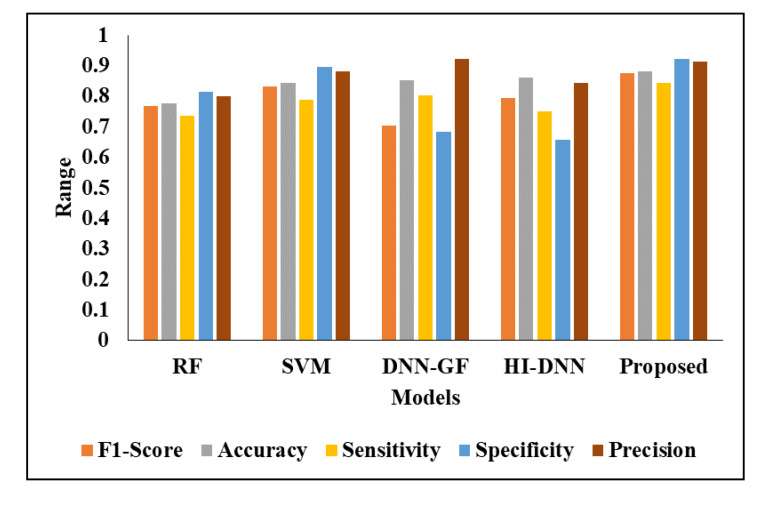
Analysis of the proposed model in terms of various metrics.

**Figure 8:**
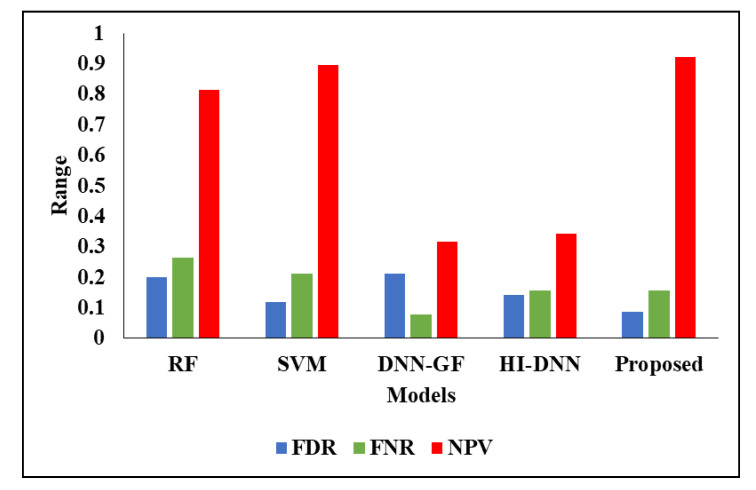
Graphical representation of the proposed model.

When comparing with existing techniques, the proposed model achieved better performance than existing techniques such as RF, SVM, DNN-GF [17] and HI-DNN [21]. The existing techniques are implemented in our dataset and system, then results are averaged in Table 4. Initially, without considering the segmentation process, all techniques identified the tumours, which shows poor performance. For instance, the proposed model has 88% of accuracy, 92% of specificity, 92% of NPV and existing techniques achieved nearly 89% of NPV, 69% to 87% of specificity and 86% of accuracy. Figure 7 and Figure 8 presents the graphical analysis of the proposed model with existing techniques. In the next analysis, all techniques are tested with segmentation and it is provided in Table 5. 

**Figure 9:**
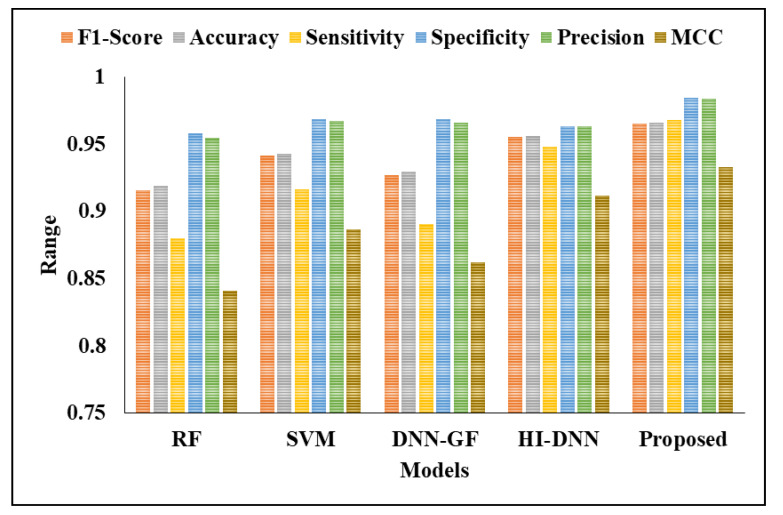
Analysis of the proposed model with existing techniques.

**Figure 10:**
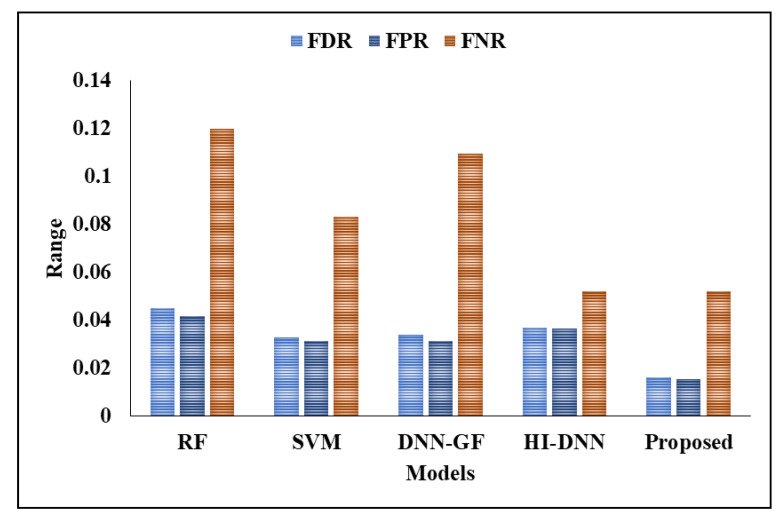
Graphical representation for error analysis.

**Figure 11:**
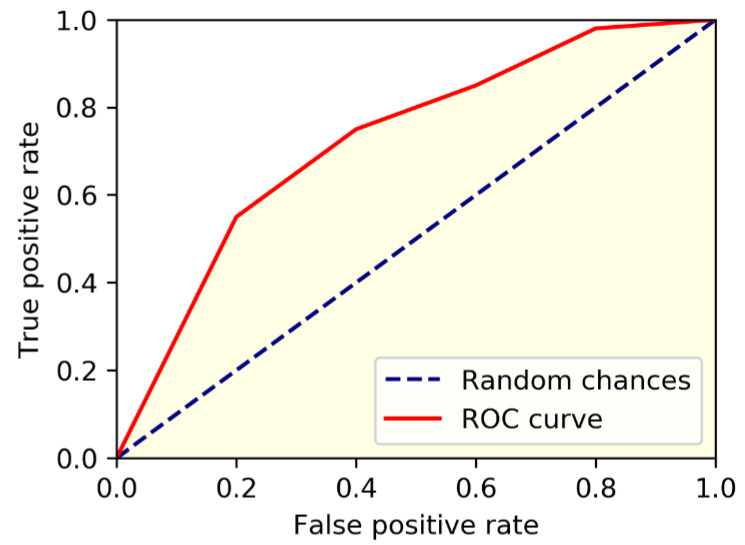
ROC analysis of the proposed model.

When segmentation techniques are considered, the results for even existing techniques have better performance. For instance, the existing techniques achieved nearly 92% to 95% of accuracy and the proposed model achieved 96% of accuracy. The reason for better performance is that the kernels are optimally selected by using COA and segmentation of liver is considered. The existing techniques did not use any optimization models for optimal parameter selection and achieved poorer performance than the proposed model. The FPR is high for RF, where other existing techniques has 0.03 and the proposed model has 0.01. Figure 9 and Figure 10 presents the graphical analysis for various metrics. The analysis of ROC for the proposed model is presented in Figure 11.

### 4.3. Cross-Valdiation Analysis

Here, the analysis is carried out on various k-fold cross validations. Table 6 presents the analysis of the proposed model with existing techniques by considering different cross validations.

The above table presents the different ratio of training images and testing images, with cross-validation analysis. It is shows that the proposed model achieves nearly 96% to 98% for 80-20, 90-10, and cross-validation, but the same model achieved only 93.45% accuracy, when the training is 70% and testing is 30%. This shows that the splits play an important role for classification accuracy. The existing techniques such as HI-DNN and DNN-GF achieved nearly 93% to 96% of accuracy for 90-10 split, 80-20 split, and cross-validation, where those models achieved only 91% of accuracy, when the split is 70-30. For the analysis of coot optimization in the proposed model, Table 7 provides the experimental analysis.

The computational cost of the proposed model is lower (0.71 GB) compared to the existing methods, and it also requires less memory size (237.89 MB). However, the existing RF model has 1.18 G of computation cost and uses 289.11MB of memory size to process the identification. This leads to poor classification performance and the main reason is that a trained RF requires high memory for storage, due to the need for retaining the information from several hundred individual trees. In addition, the requirement of training time and testing time of various algorithms is depicted in Table 8.

From the above experiments, it is clearly proven that the proposed model has less training time and testing time of input liver images. When compared with all techniques, RF has high training time and testing time due to hundreds of trees, which is previously explained in Table 6. The other existing models such as DNN-GF and HI-DNN has nearly 2300 s of training time and 14 s for testing the input image.

### 4.4. Analysis of Proposed Classifier Model on Silver07 Dataset

Table 9 compares the proposed model to existing techniques for the Silver07 dataset with regards to segmentation.

In the analysis of accuracy, the existing ML achieved nearly 78/5 to 83%, DNN-GF achieved 80%, HI-DNN model achieved 83%, and the proposed model achieved 87.09%. The reason for better performance is that the ELM’s weight parameters are optimally selected by COA, where existing techniques did not consider parameter selection operation. In the analysis of FPR, the RF has 0.12, SVM has 0.18, DNN-GF and HI-DNN have 0.06, and the proposed ELM has 0.05. The sensitivity and specificity of the proposed model achieved nearly 99%, where the existing models such as RF has nearly 80–87%, SVM has 73–81%, and HI-DNN achieved nearly 93% to 98%. The importance of COA in ELM is tested with both datasets, which is provided in Table 10.

In the first dataset (3DIRCADb1), the proposed model achieved 87% without COA and the same model achieved 96% with COA. This analysis shows the importance of COA in ELM’s parameter selection. Likewise, without COA, the proposed ELM achieved 68% of F-measure, 83% of sensitivity, 96% of specificity, and 95% of precision. The same ELM model achieved 96% of F-measure, sensitivity, and it has 98% of specificity and precision, when it is tested with COA. In the second dataset, the ELM has 81% of accuracy, 75% of F1-score, 95% of sensitivity, 98% of specificity, and 94% of precision without COA. However, when the ELM was tested with COA, it achieved 87% of accuracy, 84% of F1-score, 99% of sensitivity, 99% of specificity, and 99% of precision. In the analysis of FPR, the proposed model has 0.17 on first dataset and 0.19 for second dataset without COA. However, the same ELM achieved 0.15 of FPR on first dataset and second dataset, when it tested with COA.

## 5. Conclusions

We present a deep learning-based communicating system with high accuracy for segmenting tumours from liver pictures. To encode user communications to direct CNN for a decent initial subdivision, a new context-aware approach was presented. In addition to the encoding technique, we also suggested a powerful refining strategy to boost the precision of the segmentation outcomes. Segmentation is an important goal for models built using deep learning, and this framework is intended to help achieve that goal. The results of our experiments on a broad variety of input photos demonstrate that (1) our inner margin points and EGD architecture provides superior accuracy and efficiency learning-based interactive segmentation tools. In addition, the suggested framework is very generalizable with regards to liver tumour identification. As an annotation tool, it might be used to produce segmentation masks quickly and precisely for various objects. As a future work, the classification accuracy could be improved by introducing the deep learning model in the research work for classification instead of a machine learning model.

## Figures and Tables

**Table 1:** Detailed info of 3Dircadb. (F: Female; M: Male).

**S. No**	**Gender**	**Voxel Dimensions**	**Slices**	**Tumours**
1	F	0.57 × 0.57 × 1.6	129	7
2	F	0.78 × 0.78 × 1.6	172	1
3	M	0.62 × 0.62 × 1.25	200	1
4	M	0.74 × 0.74 × 2.0	91	7
5	M	0.78 × 0.78 × 1.6	139	0
6	M	0.78 × 0.78 × 1.6	135	20
7	M	0.78 × 0.78 × 1.6	151	0
8	F	0.56 × 0.56 × 1.6	124	3
9	M	0.87 × 0.87 × 2.0	111	1
10	F	0.73 × 0.73 × 1.6	122	8
11	M	0.72 × 0.72 × 1.6	132	0
12	F	0.68 × 0.68 × 1.0	260	1
13	M	0.67 × 0.67 × 1.6	122	20
14	F	0.72 × 0.72 × 1.6	113	0
15	F	0.78 × 0.78 × 1.6	125	2
16	M	0.70 × 0.70 × 1.6	155	1
17	M	0.74 × 0.74 × 1.6	119	2
18	F	0.74 × 0.74 × 2.5	74	1
19	F	0.70 × 0.70 × 4.0	124	46
20	F	0.81 × 0.81 × 2.0	225	0

**Table 2:** Segmentation Results on 3DIRCADb1 dataset.

Method	Dice Score	Jaccard	Accuracy	Specificity	Sensitivity	SVD
GW-CTO [21]	67.5 *± 27*.8%	56.0 *±* 30.7%	92 *±* 3.8%	70.1 *±* 29.6%	64.8 *±* 32.2%	0.33
Proposed	77.11 *±* 21.0%	67.8 *±* 26.9%	93 *±* 3.7%	79.16 *±* 20.56%	76.03 *±* 24.56%	0.23

**Table 3:** Segmentation Results on Silver07 Dataset.

Method	Dice Score	Jaccard	Accuracy	Specificity	Sensitivity	SVD
GW-CTO [21]	70.7 ± 24.9 %	69.5 ± 34.6%	91 ± 3.9%	73.5 ± 27.6%	67.6 ± 33.26	0.25
Proposed	77.54 ± 21.5%	65.5 ± 32.5%	92 ± 3.9%	80.36 ± 4.6%	77.51 ± 25.66	0.22

**Table 4:** Classification results without segmentation.

Metrics	RF	SVM	DNN-GF	HI-DNN	Proposed
FDR	0.2	0.11765	0.2105	0.14211	0.085715
F1-Score	0.76712	0.83333	0.70345	0.79355	0.87672
Accuracy	0.77642	0.84211	0.85174	0.86241	0.88258
Sensitivity	0.73684	0.78947	0.80263	0.75	0.84212
Specificity	0.81579	0.89474	0.68421	0.65789	0.92105
FNR	0.26316	0.21053	0.078947	0.15789	0.15789
NPV	0.81579	0.89474	0.31579	0.34211	0.92105
Precision	0.8	0.88235	0.92105	0.84211	0.91429
FPR	0.18421	0.10526	0.89655	0.80645	0.078947
MCC	0.55436	0.68803	0.77612	0.72464	0.76555

**Table 5:** Classification results with segmentation.

Metrics	RF	SVM	DNN-GF [17]	HI-DNN [21]	Proposed
FDR	0.045198	0.032967	0.033898	0.037037	0.016216
F1-Score	0.91599	0.94118	0.92683	0.95539	0.96553
Accuracy	0.91927	0.94271	0.92969	0.95573	0.96615
Sensitivity	0.88021	0.91667	0.89063	0.94892	0.96792
Specificity	0.95833	0.96885	0.96875	0.96354	0.98438
Precision	0.9548	0.96703	0.9661	0.96296	0.98378
FPR	0.041667	0.03125	0.03125	0.036458	0.01562
FNR	0.11979	0.083333	0.10948	0.052083	0.05208
NPV	0.95833	0.96875	0.96875	0.96354	0.98438
MCC	0.84111	0.88662	0.86201	0.91157	0.93291

**Table 6:** Analysis for Cross-Validation.

Model	90-10 Split	80-20 Split	70-30 Split	Cross Validation
Proposed	98.9	96.3	93.45	96.65
HI-DNN	97.4	95.4	91.0	95.57
DNN-GF	96.7	94.5	90.2	92.96
SVM	94.5	92.6	88.3	94.27
RF	93.3	91.4	86.1	91.92

**Table 7:** Computation Cost and Memory Analysis of Different Techniques.

Model	Size (MB)	MACs (G)
Proposed	237.89	0.71
HI-DNN	461.10	1.03
DNN-GF	370.88	1.14
SVM	270.87	1.13
RF	289.11	1.18

**Table 8:** Training and testing time analysis.

Model	Training Time (s)	Testing Time (Image/s)
RF	2804	38.7
SVM	2705	17.6
DNN-GF	2506	15.9
HI-DNN	2011	13.1
Proposed model	2103	10.3

**Table 9:** Classification Results with Segmentation.

Metrics	RF	SVM	DNN-GF [17]	HI-DNN [21]	Proposed
FDR	0.14286	0.21429	0.25625	0.083355	0.06587
F1-Score	0.82759	0.75862	0.69565	0.85631	0.84615
Accuracy	0.83871	0.77419	0.80645	0.838771	0.87097
Sensitivity	0.80000	0.73333	0.85204	0.93752	0.99368
Specificity	0.87525	0.8125	0.99965	0.98752	0.99787
Precision	0.85714	0.78571	0.99654	0.91667	0.99368
FPR	0.12525	0.1875	0.06548	0.0654	0.0587
FNR	0.22221	0.26667	0.46667	0.26651	0.21488
NPV	0.8741	0.8125	0.98756	0.9375	0.56845
MCC	0.67783	0.54812	0.60911	0.6825	0.76594

**Table 10:** Comparative analysis of the proposed ELM with and without COA.

Dataset	3DIRCADb1 Dataset		Silver07	
Metrics	Without COA	With CoA	Without COA	With CoA
FDR	0.057497	0.016216	0.098421	0.06587
F1-Score	0.68794	0.96553	0.75297	0.84615
Accuracy	0.87668	0.96615	0.81067	0.87097
Sensitivity	0.83458	0.96792	0.95485	0.99368
Specificity	0.96568	0.98438	0.98365	0.99787
Precision	0.95584	0.98378	0.94658	0.99368
FPR	0.17278	0.1562	0.19780	0.15687
FNR	0.15684	0.05208	0.86925	0.21488
NPV	0.70654	0.98438	0.46825	0.56845
MCC	0.61545	0.93291	0.41892	0.76594

## Data Availability

The datasets used and/or analysed during the current study are available from the corresponding author upon reasonable request.
